# Analytical correction of an extension of the “MU Fraction Approximation” for Varian enhanced dynamic wedges

**DOI:** 10.4103/0971-6203.62195

**Published:** 2010

**Authors:** Michael S. Gossman, Subhash C. Sharma

**Affiliations:** Tri-State Regional Cancer Center, Medical Physics Section, 706 23^rd^ Street, Ashland, Kentucky 41101, USA; 1 Parkview Comprehensive Cancer Center, Radiation Oncology Department, 11141, Parkview Plaza Drive, Fort Wayne, Indiana 46845, USA

**Keywords:** Correction, dynamic, enhanced dynamic wedges, enhanced, wedge

## Abstract

The most common method to determine enhanced dynamic wedge factors begins with the use of segmented treatment tables. These segmental dose delivery values set as a function of upper jaw position are the backbone of a calculation process coined the “MU Fraction Approximation.” Analytical and theoretical attempts have been made to extend and alter the mathematics for this approximation for greater accuracy. A set of linear equations in the form of a matrix are introduced here which correct one published extension of the MU Fraction Approximation as it applies to both symmetric and asymmetric photon fields. The matrix results are compared to data collected from a commissioned Varian Eclipse Treatment Planning System and previously published research for Varian linear accelerators. A total enhanced dynamic wedge factor with excellent accuracy was achieved in comparison to the most accurate previous research found. The deviation seen here is only 0.4% and 1.0% for symmetric and asymmetric fields respectively, for both 6MV and 18MV photon beams.

## Introduction

Enhanced dynamic wedges (EDW) have been in use for over a decade now.[1] EDW exists as a mode that can be set on a linear accelerator allowing one assigned blocking jaw to move across the radiation field during exposure. For the Varian 21EX linear accelerator used here, the wedge effect is created by moving the upper ŷ-axis jaw. The exact position of the jaw is defined at each equally spaced segment by a strict set of values. These positional values of incremental dose are data belonging to the golden segmented treatment table (S_G_).[2] Data from the S_G_ is a function of energy and field separation of the moving jaw only. There are many different wedge angles that can be created using these tables. The segmental motion of the jaw, designated for a 60° enhanced dynamic wedge, can be modified to create enhanced dynamic wedges of alternate angles.

The concept of jaw motion yielding a wedge effect is understood when considering the number of monitor units given at each segmental step while the jaw moves across the field. One side of the field is set in motion by a single moving jaw initially. On a planning system, or during the measurement process, locations beneath this side of the field get blocked the most. It therefore constitutes the heel side of the EDW. Conversely, the opposite side of the field is unblocked by the moving jaw during most of the exposure. Hence, it constitutes the toe side of the EDW. The angle of the wedge profile with respect to the fifty percent dose intensity level at a depth of 10cm defines the EDW angle. One can achieve the desired wedge angle by simply adjusting the dose delivery at each segment. This is governed by jaw motion as it moves across the radiation field. Using golden segmented treatment tables we can then arithmetically describe the resulting ratio of dose at a chosen calculation point in the field for any energy and any desired EDW angle, knowing the depth of the calculation point and the effective field size at that depth.[3]

The coined “MU Fraction Approximation” (MFA) describes the total enhanced dynamic wedge factor (EDWF) as the ratio of the monitor units required for a calculation point within the field to receive the required dose versus the monitor units required without the enhanced dynamic wedge.[4,5] The total EDWF differs from the ordinary EDWF when off-axis ratios are multiplied into it. The difficulty in determining the total EDWF mathematically increases as the field shape becomes more asymmetric, as some researchers have likewise found.[4,6] One of the most thoughtful extensions to this theory introduces a need to correct the total EDWF when the calculation point is off-axis.[1] Since then, research has proven that this extension of the MU Fraction Approximation still needs improved accuracy. This is especially true when the calculation point is not at the center of the field and when the field separation in the moving jaw axis is large.[7,8]

This article discusses how the accuracy of the extension of the MU fraction can be improved with the introduction of an additional correction factor. Although the inaccuracy in the total EDWF can be significantly large for certain field separations, a correction factor applied to the result from an extension of the MU Fraction Approximation can generate improved results. Accumulated data will be compared to Varian Eclipse Treatment Planning System data and previously successful research. Finally, a matrix of corrections will be presented that enables calculations which satisfy the accuracy required clinically in the determination of the total EDWF for any wedge angle currently available on the Varian 21EX linear accelerator.

## Materials and Methods

### MU fraction approximation

Off-axis factors are known to have primary and scatter components.[9] Variations in these components have been seen for EDW factors in comparison to open fields, differing by up to nearly 2% for the largest fields.[10] Applied off-axis EDW factors for asymmetric fields have also been experimentally studied. Researchers found that such factors do not account for off-axis primary fluence and energy variations caused by the beam flattening filter.[11] It is for these reasons that scientists have chosen the direct route of incorporating the central axis EDW factor and the associated off-axis ratio into an all inclusive total EDW factor. Consistent with published research found, all EDW factors mentioned here are total enhanced dynamic wedge factors.

Typically, hard wedges corresponding to angles 15°, 30°, 45° and 60° are provided for customers. A solution to obtain wedge effects at intermediate angles may be achieved by combining open fields to a series of exposures using any one of these wedges, which is the basis for the advent of a universal wedge. Petti and Siddon explained how a segmented treatment table (STT) for any angle of wedge can be generated using the golden segmented treatment table (S_G_) for a 60° EDW similarly.[12] However, they note that the wedge angle can be achieved without an additional wedge filter. Instead, it can be achieved using jaw motion already intact within the collimator.

The concept involves consideration of the effect jaw displacement (ΔY) has on the segmented treatment table. In essence, the total effect is a summation of adjusted segmented treatment values involving a stationary field portion and a dynamic field portion. For the stationary field portion, the segmented treatment table is given as S_G_(Y=0). The corresponding wedged portion of the field is given as S_G_(Y), which is fully dependent on the starting and ending point of the moving jaw. For any wedge angle in question, we use Equation 1 and 2 (identified below). In it, the weight variable (w) is needed to adjust the golden segmented treatment table to reflect the wedge angle (θ) one requires.[12]

(1) $$w_{60}\equiv \frac{\tan\left(\theta \right)}{\tan\left(60^{{}^{\circ}}\right)}$$

(2) $$w_{0}\equiv \left(1-w_{60}\right)$$

The moving jaw stops at a distance 0.5cm from the fixed jaw position. Denoting Y_IJ_ as the initial moving jaw setting and Y_FX_ as the fixed jaw setting, the total jaw separation is given as ΔY=−Y_IJ_+Y_FX_. The final jaw position (Ỹ_FJ_) after moving across the plane is then Ỹ_FJ_=Y_FX_−0.5. When the assigned numbers of monitor units are delivered at the same moment jaw motion stops, the segmented treatment table is said to be normalized. In this case, each segment stepped appropriately across the field with the allotted number of MUs defined by the segment table. Thus, S_G_(θ, Ỹ_FJ_) is exactly unity. The EDWF for any wedge angle may be written as a quotient of two summations, as in Equation 3.[6]

(3) $$EDWF\equiv \frac{W_{0}S_{G}\left(0\right)+W_{60}S_{G}\left(Y_{0}\right)}{W_{0}S_{G}\left(0\right)+W_{60}S_{G}\left({\tilde{Y}}_{FJ}\right)}$$

The numerator represents the fraction of monitor units used while motion is carried out from the initial position of the moving jaw to the central axis. The denominator represents the fraction of monitor units used while motion is carried out from the initial position of the moving jaw, across the center, to the final position. It is important to note that the sign of any value for Y is negative for jaw positions opposite the side of the stopping position with respect to the central axis.

### Extensions to MU fraction approximation

Gibbons extended the MU Fraction Approximation, restricting the point of calculation to be located at the geometric center of the initial open field.[1] The equation for finding geometric center is Y_0_=(Y_IJ_+Y_FX_)/2, where the sign of any value for the calculation position (Y) is likewise negative when it exists opposite the side of the stopping position with respect to the center of the initial open field. This is true regardless of whether the field is awkwardly asymmetric.

In asymmetric circumstances, the first phase of incremental jaw movement or “sweep” as it's called may have considerably more monitor units assigned than the other. Gibbons extended the original MU Fraction Approximation to reduce this problem by noting the lack of scatter dose calculated during the first sweep and the lack of increased dose from jaw transmission in the second sweep. For center of field geometry, the total EDWF was presented as Equation 4.[1]

(4) $$EDWF\equiv \frac{W_{0}S_{G}\left(0\right)+W_{60}S_{G}\left(Y_{0}\right)+W_{60}a_{1}b_{1}\alpha e^{\beta Y_{0}}\left(\frac{e^{\left(b_{-}\right)}{\tilde{Y}}_{FJ}-e^{\left(b_{-}\right)Y_{0}}}{b_{-}}+e^{2b_{1}Y_{0}}\left[\frac{e^{-\left(b+\right)}{\tilde{Y}}_{FJ}-e^{-\left(b+\right)Y_{0}}}{b_{+}}\right]\right)}{W_{0}S_{G}\left(0\right)+W_{60}S_{G}\left({\tilde{Y}}_{FJ}\right)}$$

Fit parameters (a_1_ and b_1_) are involved in his approximation of the S_G_ data. Other parameters (α and β) are used to describe the pattern of intensity fall-off just outside the photon field. The value b_±_=b_1_±β interrelates the effect of dose delivery with S_G_.

As was pointed out by Wichman, this formalism does not satisfy the need to have points outside of the center of field.[13] This may be important dosimetrically, since the central axis is blocked for several common clinical circumstances. When some point of calculation, taken somewhere other than in the center of the field, lies within the first sweep, the dose is underestimated. Further, when the point of calculation lies beyond the center of the field in the second sweep, the dose is overestimated.

Prado *et al.* experienced these problems using the ADAC Pinnacle Treatment Planning System.[3,6,14] Independent of the calculation point location, the Prado group identified a method of correcting the field size dependence empirically, by separating the total EDW factor into three terms: (1) the central axis factor; (2) a non-linear scatter correction term, which is a function of the moving jaw field length; and (3) an off-axis correction factor. Interestingly, the validation of this technique found up to 3.5% disagreement versus measurement of off-axis points located 5cm or greater with field sizes greater than or equal to 20×20 cm^2^. The addition of the third term is more cumbersome than the MU Fraction Approximation, though not limited in that technique to central axis usage only.

Wichman was concerned with this same issue regarding the MU Fraction Approximation.[13] He showed that by replacing the geometric center variable Y_0_ with the position of the point of interest Y_i_ in the third term of the numerator in Equation 4, the drawback of this required central axis point of interest limitation is removed. Using this methodology, however, forces the calculation point of interest to be the center of the initial open field.[13] No treatment planning system comparisons were done. Measurements from this EDWF research revealed within 3% accuracy for 6MV and 18MV x-rays.

Kuperman sought a remedy for the same stated limitation of the MU Fraction Approximation, permitting off-axis calculation points to be used with even better accuracy without a third term. He modified the extension equation by applying an analytical shift in the point of calculation.[7] The shifted value Ỳ for the position of any particular calculation point (Y) is found from Equation 5.

(5) $$\overset{\backslash }{Y}\equiv Y+\lambda \left(\Delta Y\right)+\mu \left(Y_{0}-Y\right)$$

Rewriting the nomenclature for consistency with Gibbons, the center of the field is again found as Y_0_ = (Y_IJ_+Y_FX_)/2. The field length is given as ΔY. The additional parameters (λ and *μ*) are scatter dose correction weights. This value for Ỳ is substituted for values Y in the extension of the MU Fraction Approximation, where the lookup values in the golden segmented treatment table can be correctly identified.[8]

The difference in the total EDW factor calculated by the MFA theory and by the shift method was reviewed by Kuperman using a symmetric field of 20×20 cm^2^ and placing the calculation point across the length of the field.[7] The calculation point was changed in 2.5 mm intervals along directions heel and toe using first the extended MU Fraction Approximation and then his shift method. After analytical tabulation, measurements were taken for theoretical validation. The shift method proved to be accurate to within 1.0-1.8% for both 6MV and 18MV beams. For 6MV specifically, the MFA theory was nearly 11% off in the heel direction and nearly 6% off in the toe direction for a 60° wedge. Similarly for 18MV, the MFA theory was nearly 6% off in the heel direction and nearly 4% off in the toe direction for a 60° wedge. Differences existing for the other wedge angles were not presented.

### Correctional matrix

This article examines the difference between the extended MFA theory and the shift method for both 6MV and 18MV photon beams of a Varian 21EX linear accelerator for 10°, 15°, 20°, 25°, 30°, 45° and 60° EDW angles. The difference plots between the two processes create a pattern of variation.

Based on the emerging pattern, a matrix of linear equations can be implemented to improve the extended MFA inaccuracy. Each equation exists as a function of only the distance from the center of the field to the point of calculation. There are separate equations for each wedge angle and energy. To correct the resulting total enhanced dynamic wedge factor produced in the extended MFA theory, one simply needs to use the resulting correction value by the solved equation specific to that energy, wedge angle and geometry. The equations introduced here may be successfully applied even when the calculation point is not forced to be located at the center of the field.

### Analysis method

A comparison between the extended MU Fraction Approximation and the shift method for the determination of the total enhanced dynamic wedge factors are introduced in this article for 18MV photon beams having a 20×20 cm^2^ field size. The plot represents the ratio EDWF_MFA_/EDWF_Shift_. The magnitude of variance described by Kuperman was again seen here for the 60° wedge, and were correctly inverted. Well over 6% difference is seen between his method and the extended MU Fraction Approximation in extreme off-axis point placements. As introduced in this research only, for other wedge angles the difference is reduced significantly. For example, using a much smaller angle of 10° for both energies, the dissimilarity is less than or equal to 1%. The difference is reduced by nearly 8% from the 60° result for 6MV at 8cm off-axis to heel. It is found that the level of disagreement between the two methods declines as the wedge angle decreases from 60° to 10°.

### Treatment planning system validation

Analytical results from the correction matrix were compared to phantom planning data accumulated from Varian Eclipse External Beam Planning Software Version 6.5, using the 7.3.10 photon pencil beam convolution algorithm.[15] The planning system was already commissioned for EDW use. Previous publications were found detailing comparisons of EDW data compiled from various planning systems to detector measurements.[10,16] Unlike these referenced planning systems, Eclipse does not require external data for Varian enhanced dynamic wedges. All the wedge information can be calculated from the golden segmented treatment tables imbedded in the algorithm.[17] A correlation between measured EDW profile data and the Eclipse Treatment Planning System has been conducted in detail.[18] The research was conducted for each wedge angle, under isocentric geometry, at a fixed water equivalent depth of 15cm. Field sizes included in the study were symmetric apertures of 5×5 cm^2^, 10×10 cm^2^, 15×15 cm^2^ and 20×20 cm^2^.[18] Analysis of data encompassing both 6MV and 18MV resulted in a maximum deviation of only 2.8% and average deviation of less than 1% overall.[18]

## Results and Discussion

**Figure 1:**
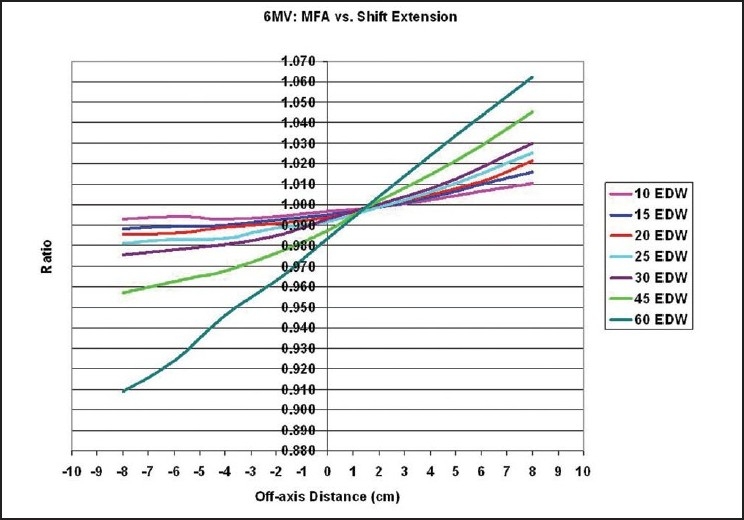
The ratio EDWFMFA/EDWFShift is plotted for Varian enhanced dynamic wedges using a 6 MV beam. For a 20×20 cm^2^ field size, the difference in the total enhanced dynamic wedge factor calculated between the two methods is plotted for all locations heel and toe, where the point of calculation is shifted away from the center of the initial open field

**Figure 2:**
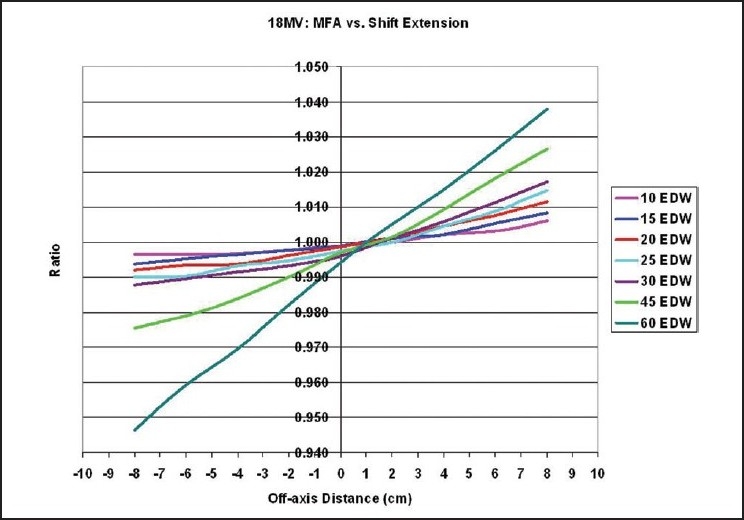
The ratio EDWFMFA/EDWFShift is plotted for Varian enhanced dynamic wedges using an 18 MV beam. For a 20×20 cm^2^ field size, the difference in the total enhanced dynamic wedge factor calculated between the two methods is plotted for all locations heel and toe, where the point of calculation is shifted away from the center of the initial open field

**Figure 3:**
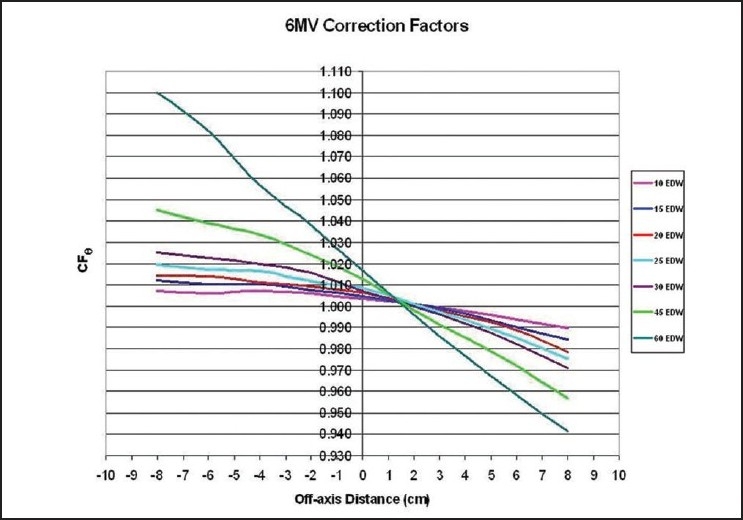
Correction factors necessary to correct the extended MU Fraction Approximation to yield shift method results is plotted for a 6 MV photon beam

**Figure 4:**
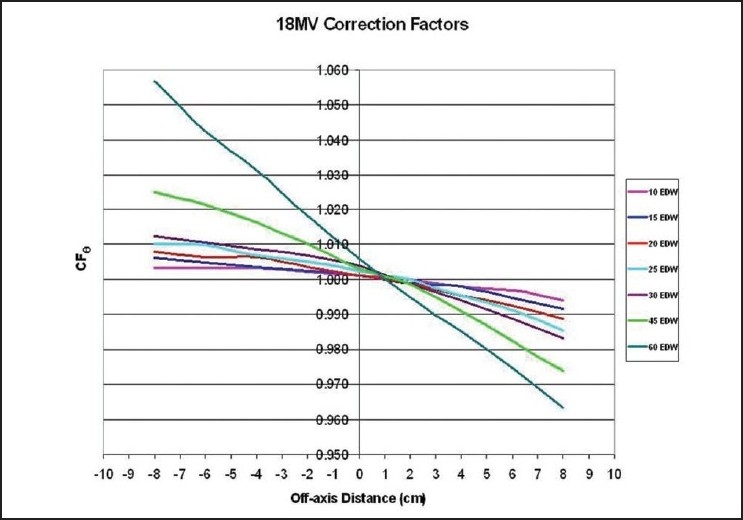
Correction factors necessary to correct the extended MU Fraction Approximation to yield shift method results is plotted for an 18MV photon beam

A comparison between the extended MU Fraction Approximation and the shift method for the determination of the total enhanced dynamic wedge factors is introduced in Figure 1 for 6MV and in Figure 2 for 18MV photon beams having a 20×20 cm^2^ field size. The form of the identified correction factors and solutions are presented in Figure 3 and Table 1 for 6MV and in Figure 4 and Table 2 for 18MV.

**Table 1:** Correction factors CFθ necessary to correct the extended MU Fraction Approximation to yield shift method results for a 6 MV photon beam

*CFθ*	*OAX (CM)*														

*EDW*	*-7*	*-6*	*-5*	*-4*	*-3*	*-2*	*-1*	*0*	*1*	*2*	*3*	*4*	*5*	*6*	*7*
10	1.008	1.008	1.007	1.006	1.005	1.004	1.002	1.001	1.000	0.999	0.998	0.997	0.996	0.995	0.994
15	1.014	1.012	1.010	1.009	1.007	1.005	1.004	1.002	1.000	0.999	0.997	0.995	0.993	0.992	0.990
20	1.018	1.015	1.013	1.011	1.009	1.007	1.004	1.002	1.000	0.998	0.996	0.993	0.991	0.989	0.987
25	1.022	1.019	1.017	1.014	1.011	1.009	1.006	1.003	1.001	0.998	0.995	0.992	0.990	0.987	0.984
30	1.028	1.024	1.021	1.018	1.014	1.011	1.007	1.004	1.001	0.997	0.994	0.990	0.987	0.984	0.980
45	1.047	1.041	1.036	1.030	1.024	1.019	1.013	1.008	1.002	0.996	0.991	0.985	0.980	0.974	0.968
60	1.089	1.079	1.069	1.059	1.049	1.039	1.029	1.019	1.009	0.998	0.988	0.978	0.968	0.958	0.948

**Table 2:** Correction factors CFθ necessary to correct the extended MU Fraction Approximation to yield shift method results for a 18 MV photon beam

*CFθ*	*OAX (CM)*														

*EDW*	*-7*	*-6*	*-5*	*-4*	*-3*	*-2*	*-1*	*0*	*1*	*2*	*3*	*4*	*5*	*6*	*7*
10	1.004	1.004	1.003	1.003	1.002	1.001	1.001	1.000	1.000	0.999	0.998	0.998	0.997	0.997	0.996
15	1.006	1.006	1.005	1.004	1.003	1.002	1.001	1.000	0.999	0.998	0.997	0.997	0.996	0.995	0.994
20	1.009	1.007	1.006	1.005	1.004	1.003	1.001	1.000	0.999	0.998	0.997	0.995	0.994	0.993	0.992
25	1.011	1.010	1.008	1.007	1.005	1.004	1.002	1.001	0.999	0.998	0.996	0.995	0.993	0.992	0.990
30	1.013	1.012	1.010	1.008	1.006	1.004	1.003	1.001	0.999	0.997	0.995	0.994	0.992	0.990	0.988
45	1.025	1.022	1.018	1.015	1.012	1.009	1.006	1.002	0.999	0.996	0.993	0.990	0.986	0.983	0.980
60	1.049	1.043	1.037	1.031	1.026	1.020	1.014	1.008	1.002	0.997	0.991	0.985	0.979	0.973	0.968

Upon inspection, one notes the strong linearity in each plot versus wedge angle. Mathematical fit analysis, applied from this supposition, revealed a matrix of linear equations which can be used and applied to the end result of the MFA theory for acceptable results. The matrices are presented below where 6MV solutions are shown as Equation 6 and 18MV solutions are shown as Equation 7.

(6) $$\left[\begin{array}{l} CF_{10^{{}^{\circ}}}\\ CF_{15^{{}^{\circ}}}\\ CF_{20^{{}^{\circ}}}\\ CF_{25^{{}^{\circ}}}\\ CF_{30^{{}^{\circ}}}\\ CF_{45^{{}^{\circ}}}\\ CF_{60^{{}^{\circ}}} \end{array}\right]≅\left[\begin{array}{ll} -0.0011 & 1.0013\\ -0.0017 & 1.0019\\ -0.0022 & 1.0022\\ -0.0027 & 1.0032\\ -0.0034 & 1.0040\\ -0.0056 & 1.0075\\ -0.0101 & 1.0186 \end{array}\right]\left[\begin{array}{l} \delta Y\\ 1 \end{array}\right]$$

(7) $$\left[\begin{array}{l} CF_{10^{{}^{\circ}}}\\ CF_{15^{{}^{\circ}}}\\ CF_{20^{{}^{\circ}}}\\ CF_{25^{{}^{\circ}}}\\ CF_{30^{{}^{\circ}}}\\ CF_{45^{{}^{\circ}}}\\ CF_{60^{{}^{\circ}}} \end{array}\right]≅\left[\begin{array}{ll} -0.0006 & 1.0002\\ -0.0009 & 1.0001\\ -0.0012 & 1.0001\\ -0.0015 & 1.0007\\ -0.0018 & 1.0008\\ -0.0032 & 1.0024\\ -0.0058 & 1.0081 \end{array}\right]\left[\begin{array}{l} \delta Y\\ 1 \end{array}\right]$$

Each matrix consists of a set of linear equations, where CF_q_ is the correction factor and δY is the displaced distance of the point of calculation from the center of the field. The value δY is given as δY=Y_POI_−Y_0_. Each linear equation has the form CF_θ_=m(δY)+b, where new parameters involved are simple linear constants (m and b) identifiable within the form of each matrix. Thus, the correction factor depends only on the point-of-interest separation from field center. Tables 1 and 2 show CF_θ_ in numerical form for 6MV and 18MV respectively.

For example, for a 6MV beam involving the 60° EDW, the correction equation is CF_60°_=−0.0101(δY)+1.0186. With a field size having y_1_ =6cm and y_2_=10cm, the point of calculation is placed at location Y=+1cm arbitrarily off-axis from the central axis toward the toe. For a wedge-OUT mode, the displaced distance of the point of calculation located from the center of field is given as δY=(1cm)−((−10cm+6cm)/2)=3cm. The center of the field is at Y=−2cm and the calculation point is located at Y=1cm. The point of interest is thus towards the toe by 3cm with respect to the center of field. The fact that CF_60°_ is positive is a hint that one should arrive at a factor δY<1.000 due to overestimation by MFA theory in this toe direction. The result for the correction factor is then calculated as follows: CF_60°_=−0.0101δY+1.0186=−0.0101(3)+1.0186=0.988. This result can be seen equally in Figure 3 or in Table 1, at 3 cm off-axis (positive) with respect to the center of the field.

For this scenario, a value of EDWF_MFA_=0.656 is found under the MFA theory and a value of EDWF_Shift_=0.645 is found under the shift theory. The disparity between the two is +1.7%, as shown in Figure 1. Again, the extended MFA theory overestimated the resulting total EDWF in the toe direction. As a result of solving the proposed equation, one may arrive at the corrected value for the total enhanced dynamic wedge factor from EDWF_MFA_×CF_60°_ = (0.656)(0.988)=0.648. This agrees well with the shift method result for the EDWF.

Analysis of capabilities of the matrix reveal maximum difference in the shift method and this correction method to be < 0.4% for symmetric fields and < 0.8% for asymmetric fields. Handling corrections of this nature is not limited to the span of the tables and plots presented here, as the correction factor exists in analytical form. Successful results can be obtained when the point of calculation is even farther off-axis, when, correctly reviewed.

## Conclusions

The MU Fraction Approximation has some shortcomings. Certainly in this theory, points closer to the center of the field result in more accuracy.[18] Large field sizes with considerably different asymmetrical arrangements, however where larger EDW angles are used, result in errors beyond 5%.[19–22] These results are a direct consequence of the inherent definitions used initially in the theory. According to this methodology, when the jaw has only moved from the initial position to the point of calculation, the number of monitor units delivered yields the entire dose to that point. We know that there will be an additional dose given from transmission through the upper jaw as it passes over the point of calculation. An additional dose is also given to the point from the radiation arriving side-scattered and back-scattered laterally, beyond the point, as the jaw continues to move onward. This results in underestimation in the heel direction and overestimation in the toe direction.

To combat these early inaccuracies, the extension of the MU Fraction Approximation was proposed, forcing the calculation point to be located at the center of the initial open field. This improved accuracy somewhat but continues to limit the user in a clinical capacity. While involved with patient plans, one should not be so concerned with the positioning of the point of calculation exactly in the center of the initial open field. Using upper chest wall planning as an example, critical organs must be removed from the field. This is especially true during certain boost procedures elsewhere as well. The arrangement of critical organs may not accommodate placing the calculation point exactly in the center for all fields, when it is necessary to shape the field to block these structures.

To achieve a more clinically acceptable solution, one can utilize a mathematical extension of this methodology, or modify it, thereby permitting the calculation of the total enhanced dynamic wedge factor at any reasonable location in the field. Kuperman has shown his shift method to work well as a modification of the extension theory. Although the limitation for the position of the calculation point was removed, the form of the equation even longer than Equation 4 from which it was contrived. Clinically, it is difficult and extremely time consuming to produce data and calculate enhanced dynamic wedge factors from it. This stands as an extreme limitation for medical physicists to not only understand the MFA as well as the extensions of it from Gibbons, Wichman and Kuperman, but also to have the ability to use it. With the perception that none of these methods are widely used clinically for mathematical checks, most facilities will not have an in-house Physics Data Book with EDWFs and EDWOARs in them.

Here, with only one variable to contend with, δY, representing the displaced distance of the point of calculation from the center of the field, the linear equations can be simply used to determine the computational correction (CF_θ_) of the Gibbons MFA Extension, yielding the final total enhanced dynamic wedge factor (EDWF_MFA_). Further, the form of their presentation will permit rapid production of in-house Physics Data Book values rather than complex equations. The example presented in the Results and Discussion Section explains their simple use.

We have shown how the MFA extension theory can be corrected directly with highlighted accuracy. The total enhanced dynamic wedge factor can be computed to within 2%, using the correction matrix as it applies to the extended MU Fraction Approximation. Analysis of the capabilities of the matrix shows the maximum difference between the shift method and this correction method to be < 0.4% for symmetric fields and < 0.8% for asymmetric fields.

The Analytical Correction Method works significantly well for the Varian model 21EX and 6EX medical accelerators. It may work equally as well for other models which also utilize the segmented treatment tables for dynamic wedge use. Since other manufacturers make use of differently constructed and operated multi-leaf systems, this data is not recommended to be used with different accelerators. Further, the total enhanced dynamic wedge factor should always be measured prior to use in this modality. It is encouraged that facilities make use of these equations only for a magnitude check on the factor if involved in computational verification.

## References

[1] Gibbons JP (1998). Calculation of enhanced dynamic wedge factors for symmetric and asymmetric photon fields. Med Phys.

[2] (2006). Varian Medical Systems, Inc. C-Series Clinac: Enhanced Dynamic Wedge Implementation Guide.

[3] Gibbons JP, Roback DM (2004). Monitor Unit Calculations for Photons and Electrons: Report of TG-71.

[4] Klein E, Low D, Meigooni A, Purdy J (1995). Dosimetry and clinical implementation of dynamic wedge. Int J Radiat Oncol Biol Phys.

[5] Klein EE, Gerber R, Zhu XR, Oehmke F, Purdy JA (1998). Multiple machine implementation of enhanced dynamic wedge. Int J Radiat Oncol Biol Phys.

[6] Prado KL, Kirsner SM, Kudchadker RJ, Steadham RE, Lane RG (2003). Enhanced dynamic wedge factors at off-axis points in asymmetrical fields. J Appl Clin Med Phys.

[7] Kuperman VY (2005). Analytical representation for Varian EDW factors at off-center points. Med Phys.

[8] Kuperman VY (2004). A new analytical model for Varian enhanced dynamic wedge factors. Phys Med Biol.

[9] Gibbons JP, Khan FM (1995). Calculation of dose in asymmetric photon fields. Med Phys.

[10] Liu C, Waugh B, Li Z, Zhu TC, Palta JR (1997). Commissioning of the enhanced dynamic wedge on a ROCS RTP System. Med Dos.

[11] Salk JE, Röttinger EM (2000). Calculation of effective Enhanced Dynamic Wedge Factors from Segmented Treatment Tables for symmetric and asymmetric photon beams. 1-8.

[12] Petti PL, Siddon RL (1985). Effective wedge angles with a universal wedge. Phys Med Biol.

[13] Wichman BD (2003). A spreadsheet solution for off-axis, non-central enhanced dynamic wedge factors. J Appl Clin Med Phys.

[14] ADAC Laboratories. ADAC Pinnacle3 Treatment Planning System, Version 6.0.

[15] Varian Medical Systems, Inc. Varian Eclipse Treatment Planning System, Version 6.5.

[16] Miften M, Weismeyer M, Beavis A, Takahashi K, Broad S (2000). Implementation of enhanced dynamic wedge in the Focus RTP System. Med Dos.

[17] Redpath T (2003). A generic computer program for checking photon beam dose calculations. Brit J Radiol.

[18] Gossman MS, Robertson MA, Lawson RC (2007). Correlation between detector array measurements and a computer algorithm for enhanced dynamic wedge profiles. Med Dos.

[19] Liu C, Kim S, Kahler DL, Palta JR (2003). Generalized monitor unit calculation for the Varian enhanced dynamic wedge field. Med Phys.

[20] Liu C, Li Z, Palta JR (1998). Characterizing output for the Varian enhanced dynamic wedge field. Med Phys.

[21] Liu C, Zhu TC, Palta JR (1996). Characterizing output for dynamic wedges. Med Phys.

[22] Chang SX, Gibbons JP (1999). Clinical Implementation of Non-physical Wedges.

